# Early Increase in Circulating PD-1^+^CD8^+^ T Cells Predicts Favorable Survival in Patients with Advanced Gastric Cancer Receiving Chemotherapy

**DOI:** 10.3390/cancers15153955

**Published:** 2023-08-03

**Authors:** Kabsoo Shin, Joori Kim, Se Jun Park, Hyunho Kim, Myung Ah Lee, Okran Kim, Juyeon Park, Nahyeon Kang, In-Ho Kim

**Affiliations:** 1 Division of Medical Oncology, Department of Internal Medicine, Seoul St. Mary’s Hospital, College of Medicine, The Catholic University of Korea, Seoul 06591, Republic of Korea; kabsoo.shin@catholic.ac.kr (K.S.); jooriworld@gmail.com (J.K.); psj6936@catholic.ac.kr (S.J.P.); angelamd@catholic.ac.kr (M.A.L.); 2 Cancer Research Institute, College of Medicine, The Catholic University of Korea, Seoul 06591, Republic of Korea; okrane@hanmail.net (O.K.); pke1001@hanmail.net (J.P.); bluesky8473@catholic.ac.kr (N.K.); 3 Division of Medical Oncology, Department of Internal Medicine, St. Vincent Hospital, College of Medicine, The Catholic University of Korea, Seoul 06591, Republic of Korea; hori1104@naver.com

**Keywords:** platinum-based chemotherapy, advanced gastric cancer, mononuclear cells, fluorescence-activated cell sorting, progression-free survival, overall survival, durable response, duration of response, immunogenic cell death

## Abstract

**Simple Summary:**

There is a lack of biomarkers for predicting the response to chemotherapy in gastric cancer (GC). This study investigates the prognostic significance of PD-1 expression in CD8^+^ T cells in the blood of 68 patients with advanced GC receiving platinum-based chemotherapy. Patients were divided based on baseline frequencies of PD-1^+^CD8^+^ T cells and whether these frequencies increased or decreased on day 7 after treatment. While baseline frequencies of PD-1^+^CD8^+^ T cells had no impact on survival, the patients whose frequencies of PD-1^+^CD8^+^ T cells increased on day 7 from the treatment initiation showed significantly longer survival than those whose frequencies of PD-1^+^CD8^+^ T cells decreased. This difference was also reflected in the mean duration of response between the groups. These findings suggest that an early increase in frequencies of PD-1^+^CD8^+^ T cells could predict favorable prognoses and durable responses in patients with advanced GC receiving chemotherapy.

**Abstract:**

The clinical significance of PD-1 expression in circulating CD8^+^ T cells in patients with gastric cancer (GC) receiving chemotherapy remains unelucidated. Therefore, we aimed to examine its prognostic significance in blood samples of 68 patients with advanced GC who received platinum-based chemotherapy. The correlation between peripheral blood mononuclear cells, measured using fluorescence-activated cell sorting, was evaluated. Patients were divided into two groups according to the changes in PD-1^+^CD8^+^ T-cell frequencies between day 0 and 7. They were categorized as increased or decreased PD-1^+^CD8^+^ T-cell groups. The increased PD-1^+^CD8^+^ T-cell group showed longer progression-free survival (PFS) and overall survival (OS) than the decreased PD-1^+^CD8^+^ T-cell group (PFS: 8.7 months vs. 6.1 months, *p* = 0.007; OS: 20.7 months vs. 10.8 months, *p* = 0.003). The mean duration of response was significantly different between the groups (5.7 months vs. 2.5 months, *p* = 0.041). Multivariate analysis revealed that an increase in PD-1^+^CD8^+^ T-cell frequency was an independent prognostic factor. We concluded that the early increase in PD-1^+^CD8^+^ T-cell frequency is a potential predictor of favorable prognoses and durable responses in patients with advanced GC receiving chemotherapy.

## 1. Introduction

Gastric cancer (GC) is the fifth most common cancer and the fourth leading cause of cancer-related deaths worldwide [1]. Systemic chemotherapy is the mainstay treatment for unresectable/metastatic GC, and recent therapeutic strategies have evolved to include a combination of PD-1 inhibitors, alongside cytotoxic chemotherapy [2,3,4,5,6].

The PD-L1/PD-1 axis has been found to be an important mechanism that tumor cells deploy to evade T-cell immunity [7,8,9]. An effective anti-tumor immune response relies on the presentation of neoantigens to cytotoxic T cells. However, when subjected to continuous stimulation by these neoantigens, cytotoxic T cells progressively lose their effector functions and overexpress PD-1, a state referred to as exhaustion [10]. Although PD-1 can be expressed on any T cell during activation, it is frequently linked to the exhaustion of CD8^+^ T cells and poor prognosis in numerous cancers including GC [11,12,13,14,15]. Nevertheless, several studies have demonstrated that PD-1^+^CD8^+^ T cells play various roles in the tumor microenvironment (TME) and peripheral blood, and some of them still maintain their cytotoxic functionality [16,17,18,19].

Recent studies on GC have shown that PD-1 expression in tumor-infiltrating CD8^+^ T cells does not always indicate exhaustion or diminished cytokine production, as these cells can produce pro-inflammatory cytokines similar to effector T cells [17]. Associations between high PD-1^+^CD8^+^ T cell levels in TME or peripheral blood and the presence of a favorable prognosis have been established [20,21,22,23].

PD-1/PD-L1 inhibitors work by blocking the PD-1/PD-L1 pathway, thereby reinvigorating CD8^+^ T cells and promoting their proliferation and the restoration of their functions [24]. Owing to these mechanistic features, numerous studies have examined the changes in the TME and circulatory immune cells, focusing on PD-1^+^CD8^+^ T cells before and after the administration of immune checkpoint inhibitors across various types of cancer [25,26,27,28,29]. In contrast, cytotoxic chemotherapy is known to modulate the tumor immune and systemic immune environment by inducing immunogenic cell death (ICD). This process results from an increase in neoantigens, as cancer cells die due to the direct tumoricidal effect of cytotoxic agents. The latter are then taken up and presented to T cells by antigen-presenting cells (APCs), thereby enhancing both innate and adaptive immunity [30,31,32,33]. While related studies on various types of cancer, including GC, do exist in the literature, research on this topic of metastatic GC remains insufficient [30,34,35,36,37].

Meanwhile, in patients with metastatic GC, the duration of response (DOR) rate at 24 months is approximately 20% for patients receiving first-line chemotherapy and immunotherapy and about 10% for those receiving cytotoxic chemotherapy only [38]. The reason these patients demonstrate a long durable response to chemotherapy may not only be due to the direct tumoricidal effect of the cytotoxic agent, but also because strong tumor-specific immunity has been induced and maintained over a long period of time without other immunotherapy.

Currently, there are no specific biomarkers that can distinguish patients with gastric cancer who show a durable response to chemotherapy. According to the ICD model, this could be elucidated by exploring changes in subsets of T cells, which are a major component of adaptive immunity. Considering the peak response of antigen-exposed T cells within 1–2 weeks and early T-cell changes observed a week post PD-1 inhibitor administration in a previous study, it is possible that chemotherapy’s impact on systemic immunity via the tumoricidal effect could be detected early in T cells [29,39,40].

Based on the hypothesis that early changes in T cells in patients with unresectable or metastatic GC treated with cytotoxic chemotherapy may be associated with durable response and prognosis, this study aimed to investigate the relationships among T cells, clinical factors, and prognosis, focusing on circulating PD-1^+^CD8^+^ T cells.

## 2. Materials and Methods

### 2.1. Patients

This retrospective study was conducted using prospectively collected samples from our previous study on GC [41]. We retrospectively enrolled patients with untreated GC at the Catholic University of Seoul St. Mary’s Hospital between March 2019 and March 2021. The eligibility criteria were patients who are ≥20 years old, diagnosed with gastric adenocarcinoma via tissue biopsy, with unresectable advanced or recurrent/metastatic disease, treated with first-line platinum-based chemotherapy, whose blood samples were obtained on day 0 and day 7, and who provided informed consent.

All enrolled patients were treated with first-line platinum-based chemotherapy. Trastuzumab was combined to the treatment regimen for human epidermal growth factor receptor 2 (HER2)-positive GC. Blood samples were collected before (day 0) and seven days after chemotherapy (day 7).

This study was approved by the Institutional Review Board of the Catholic University of Seoul Saint Mary’s Hospital (KC18TNSI0361). All patients provided written informed consent prior to enrollment.

### 2.2. Fluorescence-Activated Cell Soring Analysis

Peripheral blood mononuclear cells (PBMCs) were isolated from Ficoll-Paque density-centered blood samples, treated with ethylenediaminetetraacetic acid, and stored in liquid nitrogen before analysis. The markers used for staining the PBMCs included CD3, CD4, CD8, CD45, CD69, Ki-67, and PD-1 (BioLegend, San Diego, CA, USA). The stained cells were acquired using a BD Biosciences Canto II (BD Biosciences, San Diego, CA, USA), and the data were analyzed using the FlowJo software (Tree Star, Ashland, OR, USA). The antibodies used in fluorescence-activated cell sorting is shown in Supplementary Table S1).

### 2.3. Statistical Analysis

The time from the start of treatment to disease progression or death from any cause was defined as progression-free survival (PFS). The time from the start of treatment to death from any cause or the last follow-up date was defined as overall survival (OS). The time from response to treatment to disease progression or death from any cause was defined as the DOR.

The treatment response was evaluated using the Response Evaluation Criteria in Solid Tumors version 1.1 [42]. The depth of response is defined as the relative change in the sum of the longest diameters of target lesions at the nadir, compared with baseline [43].

Given the absence of an established definition for a durable response, we defined it as a PFS that exceeds three times the overall patient PFS [44]. To compare the durable responses across different groups, we employed the following methods: (1) We conducted univariate analyses for OS, PFS, and DOR using the Kaplan–Meier method and compared the proportion of patients who experienced a durable response across the groups; (2) we utilized the restricted mean DOR to mitigate the limitation of DOR analyses, which only accounted for responders. The restricted mean DOR, calculated from the area under the probability-of-being-in-response curve, incorporated both the response status and duration for all the patients [45,46].

Chi-squared and Fisher’s tests for categorical variables were used to compare clinicopathological differences between the groups. Statistical associations between continuous variables were analyzed using Spearman’s correlation. The Mann–Whitney U-test was used for comparisons of non-parametric values. Paired values were compared using the non-parametric Wilcoxon matched-pair signed-rank test. Univariate and multivariate Cox proportional hazards regression models were applied to analyze the variables for survival. Any variables that demonstrated a *p*-value of less than 0.10 in the univariate analysis were then included in the multivariate model. Schoenfeld residuals were used to test the assumption of proportional hazards. Statistical significance was set at *p* < 0.05. All statistical analyses were performed using R version 4.2.2 (http://www.r-project.org (accessed on 20 February 2023)).

## 3. Results

### 3.1. Clinical Characteristics

A total of 68 patients were recruited for analysis between March 2019 and March 2021. The median OS was 13.3 months (95% confidence interval (CI) 10.3–18.3 months). The median PFS was 7.0 months (95% CI 5.4–8.4 months). A total of 62 patients with HER2-negative cancer received first-line fluoropyrimidine/platinum chemotherapy (capecitabine /oxaliplatin (*n* = 45), 5-fluorouracil (5-FU)/oxaliplatin and leucovorin (*n* = 13), S-1/cisplatin (*n* = 2), and capecitabine/cisplatin (*n* = 2)) and six patients with HER2-positive cancer received trastuzumab and capecitabine/cisplatin (*n* = 4) or 5-FU/cisplatin (*n* = 2). Eighteen patients experienced recurrence after curative surgery, and fifty had either initially metastatic or unresectable advanced GC. Four tumors were characterized by microsatellite instability (MSI), fifty-nine were microsatellite-stable, and five remained undetermined. Six tumors were Epstein–Barr virus (EBV)-positive, sixty were negative, and two remained undetermined.

Patients were divided according to the fold change in the frequency of PD-1^+^CD8^+^ T cells among CD8^+^ T cells between days 0 and 7 after chemotherapy. Twenty-eight patients with fold change ≥ 1 (increased) were categorized into the “increased-PD-1^+^CD8^+^ T-cell group”, and forty patients with fold change < 1 (decreased) were categorized into the “decreased-PD-1^+^CD8^+^ T-cell group”. Table 1 summarizes the patients’ baseline characteristics according to the groups. The increased-PD-1^+^CD8^+^ T-cell group had a greater number of patients with increased CEA level (*p* = 0.034). Other clinical variables were not different between the groups (Table 1).

HER2, EBV, and MSI status were not different between the groups (Supplementary Table S2). Their baseline PD-1^+^CD8^+^ T-cell frequencies were not different (Supplementary Figure S1).

### 3.2. Correlation between T Cells and Inflammatory Markers

We examined the correlation between the complete blood count, inflammatory markers, T cells, markers related to their activation and proliferation (CD69 and Ki-67), and PD-1. The baseline value (day 0) and fold changes observed from days 0 to 7 among the parameters were assessed.

The frequency of baseline PD-1^+^CD8^+^ T cells among CD8^+^ T cells was negatively correlated with the frequency of lymphocytes in white blood cells (Rs = −0.266, *p* = 0.028) and the frequency of T cells within lymphocytes (Rs = −0.421, *p* < 0.001).

Additionally, from days 0 to 7, the fold change in the frequency of PD-1^+^CD8^+^ T cells showed a positive correlation with the fold changes in the frequency of Ki-67^+^CD4^+^ (Rs = 0.275, *p* = 0.023) and CD69^+^CD4^+^ (Rs = 0.242, *p* = 0.047) expression in CD4^+^ T cells, as well as in platelets (Rs = −0.265, *p* = 0.029). Although not statistically significant, the aforementioned fold change also showed a positive correlation with the corresponding fold changes in Ki-67^+^CD8^+^ and CD69^+^CD8^+^ T cells (Supplementary Figure S2).

### 3.3. Baseline Frequency of PD-1^+^CD8^+^ T Cells Is Not Associated with Survival

**Figure 1:**
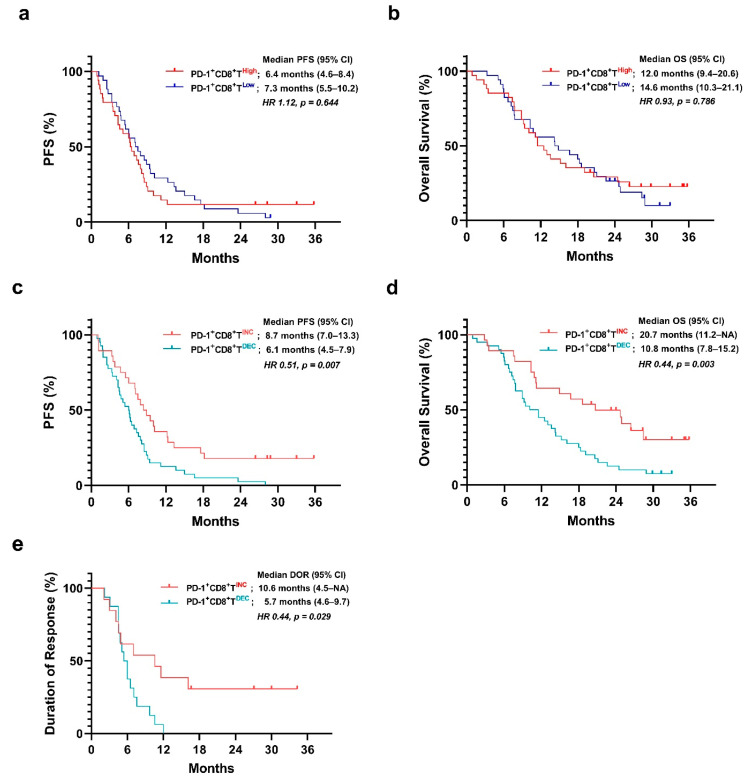
Survival difference according to baseline and early changes in the frequency of PD-1^+^CD8^+^ T cells. (**a**,**b**) Progression-free survival (PFS) and overall survival (OS) in the groups divided by the median value of the frequency of PD-1^+^CD8^+^ T cells at baseline (day 0). (**c**,**d**) PFS and OS in the groups divided by the fold change in the frequency of PD-1^+^CD8^+^ T between days 0 and 7. (**e**) Duration of response between the groups. (PD-1^+^CD8^+^T^INC^, increased PD-1^+^CD8^+^ T-cell group; PD-1^+^CD8^+^T^DEC^, decreased PD-1^+^CD8^+^ T-cell group).

Patients were classified into ‘low’ and ‘high’ groups according to the median value of baseline frequency of PD-1^+^CD8^+^ T cells they exhibited among CD8^+^ T cells. The high PD-1^+^CD8^+^ T cell group showed shorter OS than the low PD-1^+^CD8^+^ T cell group, but the difference was not statistically significant (PFS: 6.4 months vs. 7.3 months, *p* = 0.644; OS: 12.0 months vs. 14.6 months, *p* = 0.786) (Figure 1).

### 3.4. Prognostic Value of the Early Change in the Frequency of PD-1^+^CD8^+^ T Cells

The increased PD-1^+^CD8^+^ T-cell group showed significantly longer PFS than the decreased PD-1^+^CD8^+^ T-cell group (PFS, 8.7 months vs. 6.1 months; *p* = 0.007) (Figure 1). Additionally, the increased PD-1^+^CD8^+^ T-cell group showed significantly longer OS than the decreased PD-1^+^CD8^+^ T-cell group (OS, 20.7 months vs. 10.8 months; *p* = 0.003) (Figure 1).

The DOR was evaluated among the responders (*n* = 29). The increased-PD-1^+^CD8^+^ T-cell group showed a significantly longer DOR than the decreased PD-1^+^CD8^+^ T-cell group (DOR, 10.6 months vs. 5.7 months; *p* = 0.029) (Figure 1).

We established the reference point for durable response as 21.0 months, which is three times the median PFS [44]. Five out of twenty-eight patients in the increased PD-1^+^CD8^+^ T-cell group had more than 21 months of PFS compared to two out of forty patients in the decreased PD-1^+^CD8^+^ T-cell group (21-month PFS rate: 17.9% vs. 5.0%).

### 3.5. Change in CD69^+^CD8^+^ T Cells and Ki-67^+^CD8^+^ T Cells in Both Groups

**Figure 2:**
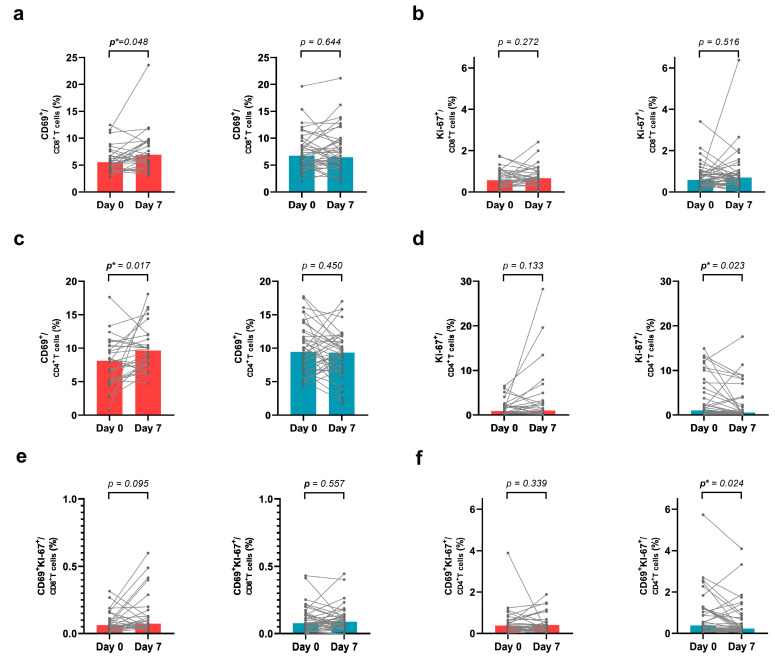
Dynamic change in CD69 and Ki-67 expression of circulating CD8^+^ (**a**,**b**,**e**) and CD4^+^ (**c**,**d**,**f**) T cells from days 0 to 7 in both the increased and decreased PD-1^+^CD8^+^ T-cell groups (red bar and blue bar). A Wilcoxon matched-pairs signed rank test was performed for statistical analysis. (**a**) The frequency of CD69 expression among the CD8^+^ T cells on day 7 was higher than that on day 0 in the increased PD-1^+^CD8^+^ T-cell group. However, no difference was found between days 0 and 7 in the decreased PD-1^+^CD8^+^ T-cell group. (**b**) No statistical difference was uncovered from Ki-67^+^CD8^+^ cells among the CD8^+^ T cells in either group. (**c**) The frequency of CD69 expression among CD4^+^ T cells increased in the increased PD-1^+^CD8^+^ T-cell group. (**d**) The frequency of Ki-67 expression among the CD4^+^ T cells decreased in the decreased PD-1^+^CD8^+^ T-cell group. (**e**,**f**) The frequency of both CD69 and Ki-67 expression among the CD8^+^ T cells (**e**) and CD4^+^ T cells (**f**). Concurrent expression of CD69 and Ki-67 among CD4^+^ T cells decreased in the decreased-PD-1^+^CD8^+^ T-cell group (*p* = 0.024). p* indicates statistical significance.

We observed dynamic changes in CD69 and Ki-67 expression in circulating CD8^+^ T cells in both the PD-1^+^ and CD8^+^ T-cell groups from days 0 to 7 for assessing the early activation and proliferation of T cells. In the increased-PD-1^+^CD8^+^ T-cell group, the frequencies of both the CD69^+^CD8^+^ and the Ki-67^+^CD8^+^ T cells among the CD8^+^ T cells increased, and the frequency of the CD69^+^CD8^+^ T cells was significantly different (*p* = 0.048). In the decreased-PD-1^+^CD8^+^ T-cell group, the frequencies of Ki-67^+^CD8^+^ and CD69^+^CD8^+^ T cells among the CD8^+^ T cells did not show significant differences (Figure 2). 

Additionally, in the increased PD-1^+^CD8^+^ T-cell group, the frequencies of both the CD69^+^CD4^+^ and the Ki-67^+^CD4^+^ T cells among the CD4^+^ T cells increased, and the frequency of the CD69^+^CD4^+^ T cells was significantly different (*p* = 0.017). In the decreased PD-1^+^CD8^+^ T-cell group, the frequency of Ki-67^+^CD4^+^ T cells among the CD4^+^ T cells was significantly decreased (*p* = 0.023) (Figure 2). Further analyses on the co-expression of Ki-67 and CD69 among CD4^+^ and CD8^+^ T cells were performed. The co-expression of CD69 and Ki-67 among CD8+T cells in the increased PD-1^+^CD8^+^ T-cell group increased between day 0 and day 7 but did not show a significant *p*-value. The co-expression of CD69 and Ki-67 among CD4^+^T cells in the decreased PD-1^+^CD8^+^ T-cell group significantly decreased (*p* = 0.024). This indicated that after the administration of cytotoxic chemotherapy, both the CD8^+^ and the CD4^+^ T cells showed increased proliferation and activation on day 7 in the increased PD-1^+^CD8^+^ T-cell group.

We further analyzed the expression of Ki-67 and CD69 in PD-1^+^CD8^+^ T cells and PD-1^+^CD4^+^ T cells. However, we found no significant results (Supplementary Figure S3).

### 3.6. Duration of Response in PD-1^+^CD8^+^ T-Cell Groups

In the increased PD-1^+^CD8^+^ T-cell group, 12 patients (42.9%) had a partial response (PR), 1 (3.6%) had a complete response (CR), 3 (17.2%) had progressive disease (PD), and 12 (42.9%) had stable disease (SD) or were not evaluable (NE).

In the decreased PD-1^+^CD8^+^ T-cell group, 16 patients (40.0%) had PR, 9 patients (22.5%) had PD, and 15 patients (37.5%) had SD/NE. The objective response rate (ORR) was higher in the increased PD-1^+^CD8^+^ T-cell group compared to the decreased PD-1^+^CD8^+^ T-cell group, but the difference was not statistically significant (ORR, 46.5% vs. 40.0%; *p* = 0.455).

**Figure 3:**
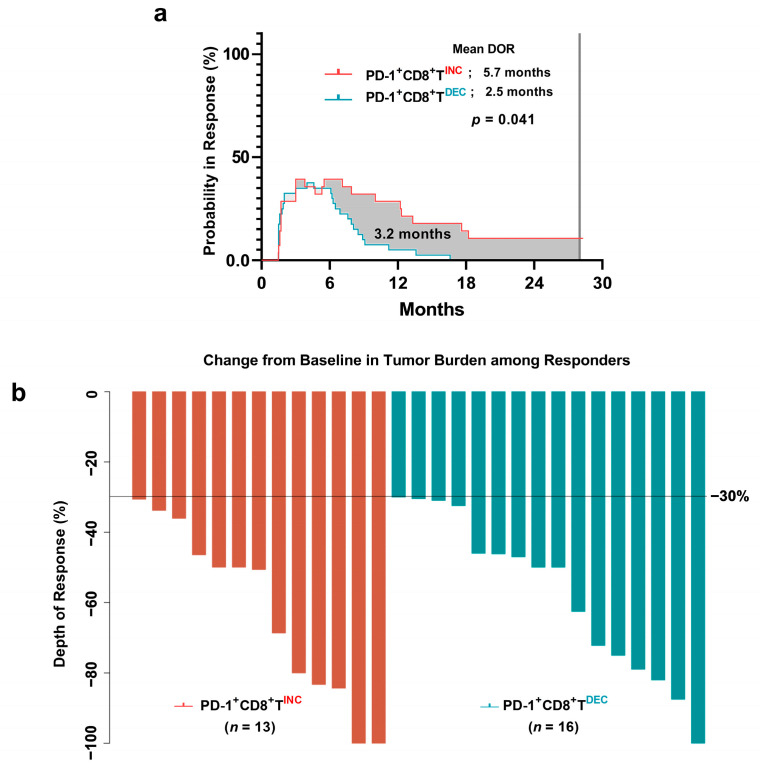
Difference in mean duration of response and depth of response in both PD-1^+^CD8^+^ T-cell groups (red bar and blue bar). (**a**) Probability of being in response (PBIR) curve. The area under the PBIR curve is the mean DOR. The increased PD-1^+^CD8^+^ T-cell group showed a longer mean duration of response than the decreased PD-1^+^CD8^+^ T-cell group. (**b**) Waterfall plot among responders from both groups. DOR, duration of response; PD-1^+^CD8^+^T^INC^, increased PD-1^+^CD8^+^ T cell-group; PD-1^+^CD8^+^T^DEC^, decreased PD-1^+^CD8^+^ T cell-group.

The mean DOR was evaluated among all the patients. The mean DOR for the increased and decreased PD-1^+^CD8^+^ T cell groups was 5.7 and 2.5 months, respectively. The difference in mean DOR was 3.2 months (95% CI 0.13–6.2 months; *p* = 0.041) (Figure 3). 

### 3.7. Difference in Depth of Response between the PD-1^+^CD8^+^ T-Cell Groups

The depth of response was not significantly different between the groups via a Mann–Whitney test (62.6% vs. 57.6%; *p* = 0.572). Although the response was achieved with cytotoxic chemotherapy, the DOR and mean DOR were significantly different owing to the early change in PD-1^+^CD8^+^ T cells (Figure 1 and Figure 3). The degree of response was not significantly associated with DOR (Figure 3).

### 3.8. Univariate and Multivariate Survival Analyses

In univariate analysis, the number of metastatic sites (≥2), a high neutrophil-to-lymphocyte ratio, and an increase in PD-1^+^CD8^+^ T-cell frequency were prognostic factors associated with OS. After adjusting for covariates, multivariate analysis showed that the number of metastatic sites (≥2) (hazard ratio (HR) 2.37, 95% CI 1.26–4.44, *p* = 0.007) and increase in PD-1^+^CD8^+^ T-cell frequency (HR 0.51, 95% CI 0.29–0.92, *p* = 0.025) were independent prognostic factors for OS (Table 2).

In the PFS analysis, the number of metastatic sites (≥2) (HR 2.55, 95% CI 1.34–4.86, *p* = 0.004) and increase in PD-1^+^CD8^+^ T-cell frequency (HR 0.57, 95% CI 0.33–0.98, *p* = 0.041) were independent prognostic factors for PFS (Table 2).

In the DOR analysis (*n* = 29), HER2, the number of metastatic sites, NLR, and increase in PD-1^+^CD8^+^ T-cell frequency showed *p*-values < 0.1 in the univariate analysis, but none of them showed independent prognostic power in the multivariate analysis (Supplementary Table S3).

### 3.9. Survival Analyses Stratified by Number of Metastatic Sites

We stratified all patients into two subgroups according to the number of metastatic sites (<2 vs. ≥2), which is an independent prognostic factor for both PFS and OS. Within each subgroup, we analyzed the survival difference between the PD-1^+^CD8^+^ T-cell groups. 

In the subgroup with more than two metastatic sites (*n* = 23), the increased PD-1^+^CD8^+^ T-cell group (*n* = 6) consistently showed longer survival compared to the decreased PD-1^+^CD8^+^ T-cell group (*n* = 17) in terms of PFS, OS, and DOR. However, statistical significance was not achieved. In the subgroup with fewer than two metastatic sites (*n* = 45), the increased PD-1^+^CD8^+^ T-cell group (*n* = 22) showed significantly longer survival in terms of PFS, OS, and DOR (PFS: 9.6 months vs. 7.9 months, *p* = 0.050; OS: 26.4 months vs. 14.2 months, *p* = 0.011; DOR: 11.6 vs. 6.8 months, *p* = 0.040). The significant survival differences between the PD-1^+^CD8^+^ T-cell groups in all patients predominantly appeared to be due to the difference seen in the subgroup with fewer than two metastatic sites (Supplementary Figure S4 and Table S4).

## 4. Discussion

In this study, we investigated the correlation between circulatory immune cells and immune-related markers at baseline (day 0) and day 7 in patients with advanced GC who received first-line platinum-based chemotherapy. We examined the prognostic and predictive values of baseline levels and changes between days 0 and 7. An increased frequency of PD-1^+^CD8^+^ T cells between days 0 and 7 demonstrated prognostic value for both OS and PFS. Additionally, when comparing the DOR and mean DOR, an increased frequency of PD-1^+^CD8^+^ T cells was associated with a longer response.

Several studies have been conducted to elucidate the prognostic significance of PD-1^+^CD8^+^ T cells present in the TME of GC; however, their results are conflicting [11,17,23,47]. Choo et al. analyzed tissues from 350 patients with stage I–IV GC and found a strong correlation between PD-1^+^CD8^+^ tumor-infiltrating lymphocytes (TILs) and the expression of Ki-67 and Granzyme B. Their findings highlight the association between the presence of PD-1^+^CD8^+^ TIL and improved prognosis [23]. Park et al. also showed that PD-1 expression in CD8 ^+^ TIL is associated with other immune checkpoint molecules (LAG3 and TIM) and better survival [47]. Shen et al. analyzed 50 patients with GC who underwent surgery and showed that PD-1^+^CD8^+^ TIL had an equivalent function to PD-1^-^CD8^+^ TIL, but they found no correlation with survival [17]. In contrast, Yu et al. showed that intratumoral PD-1^+^CD8^+^ T cells are associated with chemoresistance to adjuvant treatment and poor survival in GC [11].

Saito et al. argued that baseline circulating PD-1^+^CD8^+^ T cells were a negative predictor of resectable GC [48]. However, in this study, we were unable to confirm the impact of the frequency of PD-1^+^CD8^+^ T cells at baseline on prognosis, and we could not identify differences in clinical factors among groups distinguished by the level of baseline PD-1^+^CD8^+^ T cells except for the difference in sex distribution (Supplementary Table S5). It should be noted that our study only targeted patients receiving palliative chemotherapy, whereas Saito et al.’s study involved 72 patients with stage I–III GC.

In this study, an increase in the frequency of circulating PD-1^+^CD8^+^ T cells from baseline to day 7 after chemotherapy was strongly correlated with prognosis. Our study also showed that a week after chemotherapy, the proportion of CD69^+^CD8^+^ cells among CD8^+^ T cells, as well as CD69^+^CD4^+^ and Ki-67^+^CD4^+^ cells among CD4^+^ T cells, was also increased in the increased PD-1^+^CD8^+^ group but not in the decreased PD-1^+^CD8^+^ group. Considering the significantly longer survival and durable response observed in this group, these changes suggest that the systemic effects of chemotherapy activate circulating CD8^+^ and CD4^+^ T cells and allow them to proliferate. Thus, PD-1^+^CD8^+^ T cells might be among those activated CD8^+^ T cells which have undergone proliferation. Considering the fact that circulatory immune cells partially reflect the TME, the observed changes can be explained by the ICD model. The cytotoxic effect of the platinum-based combination may increase tumor-specific, possibly functional CD8^+^ T cells in patients with a long durable response.

Interestingly, our study found no significant difference in the response rates between groups with and without an increased frequency of PD-1^+^CD8^+^ cells. However, there was a significant difference in the DOR, and the significant difference in survival was largely attributed to the differences among responders. Similarly, Choo et al. showed that in 58 patients who received first-line platinum and fluoropyrimidine doublet as palliative treatment, patients with high PDCD1 and CD8A expression had longer PFS and OS, albeit not related to the response [23,49]. This suggests that even if the cytotoxic effect of chemotherapy provides a similar reduction effect on the tumor, other factors beyond cytotoxicity contribute to the durable effect of chemotherapy, which may be associated with increased tumor-specific immunity, represented by high circulating PD-1^+^CD8^+^ T cells levels on day 7.

There is no clear definition of ‘durable response’; however, our study has adopted the concept previously proposed by Pons-Tostivint and colleagues [44]. They defined a durable response as a PFS that exceeded three times the median PFS of all patients treated with the same drugs. Considering our cohort was treated with the platinum-based regimens, it amounted to 21.0 months in our study (median PFS of all cohort; 7.0 months). Using this criterion, we observed a difference in the 21-month PFS rate between two groups distinguished by changes in PD-1^+^CD8^+^ T cells. Additionally, the restricted mean DOR test is a composite and useful analytical method; it captures the essence of tumor response and DOR by including not only the responders within the group but also the non-responders in the analysis [50]. Using this method, we demonstrated a significant difference in the mean DOR between the two groups. Combining these significant results obtained through statistical methods and the findings of this study suggesting that early changes in PD-1^+^CD8^+^ T cells reflect immune modulation, we suggest that changes in PD-1^+^CD8^+^ T cells are a potential predictor of durable response in patients with GC receiving chemotherapy.

Numerous studies have reported that cancer therapies induce immune modulation in TME. Most of them have interpreted changes observed in surgical tissues after administering chemotherapy for 1–2 cycles or more or have been conducted by measuring circulatory immune cells. However, studies elucidating the prognostic significance of circulatory immune cells measured on day 7 after chemotherapy remain scarce [36,37,51].

To understand the changes in immune cells on day 7, one must first consider the effects of cytotoxic chemotherapy on rapidly growing cells, particularly blood cells of the lymphoid lineage. In this study, we confirmed that there was no significant difference in total white blood cell and lymphocyte counts between days 0 and 7 (Supplementary Figure S5). Furthermore, after chemotherapy, cell death in tumor cells is observed as soon as the response between chemotherapy and the tumor begins. From this point onward, neoantigens produced by the tumor interact with immune cells in the TME. Considering that the T-cell response generally peaks within 1–2 weeks following antigen exposure, the changes in immune cells observed on day 7 seem to occur in a sufficient timeframe for cancer cell response to chemotherapy to manifest and be reflected in systemic immune cells [39,40].

Another consideration is the administration of steroids, which have an immunosuppressive effect, within the chemotherapy regimen. However, considering that steroids were administered for less than four days as per our center’s protocol, their influence on day 7 might be limited. Most importantly, considering the final finding that various immune cells reflected activation/proliferation and had a significant impact on prognosis, day 7 appears to be an appropriate time point for observing immune cell activation as a result of the possible immunomodulatory effects of cytotoxic chemotherapy.

The early increase in PD-1^+^CD8^+^ T cells due to cytotoxic chemotherapy, as revealed in this study, signifies active interaction among chemotherapy, the tumor, and the immune system. This does not only reflect the tumor and TME situation but also serves as an indicator of the systemic immune state, which may not be easily inferred from the local tumor and TME in a systemically advanced GC. Moreover, this aspect is not reflected by radiographic evaluations that measure the physical indicators of the tumor itself. Additionally, it would be meaningful to measure the host response rather than the tumor response to treatment. As shown in this study, such indicators have higher predictive power than any other factor measured at baseline. 

This study has several limitations. First, as a retrospective study, it is important to acknowledge that inherent limitations such as selection bias and evaluation bias are inevitable. Additionally, while detailed analyses were performed on days 0 and 7, the subsequent samples lacked sufficient analysis. This limitation indicates the need for further investigation in this area. In addition, the analysis of PD-1^+^CD8^+^ cells in tissues, detailed subdivision consisting of the increased proportion of PD-1^+^CD8^+^ T cells, including tumor-specific T cells, and their functional analysis were not carried out. Further validation in a larger patient population is required to understand the significance of this increase in circulating PD-1^+^CD8^+^ cells.

In this study, six patients with HER2-positive GC were included. HER2-positive GC exhibits a distinct tumor biology compared to HER2-negative GC, and trastuzumab is administered in conjunction with platinum-based chemotherapy. Thus, they should be separately categorized and analyzed. EBV-positive GC (*n* = 6) and MSI GC (*n* = 4) should also be separately identified and analyzed as unique subtypes, yet due to the limited number of patients, such thorough analysis could not be fully performed. However, these groups did not demonstrate any difference in the frequency of PD-1^+^CD8^+^ T cells compared to other patients. There was no significant difference observed in the EBV, MSI, and HER2 status between the groups with increased and decreased frequencies of PD-1^+^CD8^+^ T cells. Consequently, it is considered that their influence on this study is limited. Further research should perform more comprehensive analysis on these subgroups with a larger patient cohort.

Considering the mechanism of PD-1 inhibitors, early changes in PD-1^+^CD8^+^ cells due to ICD may be related to the response to PD-1 inhibitors. Further research is required to determine whether the changes in PD-1^+^CD8^+^ cells retain their predictive value when chemotherapy is combined with PD-1 inhibitors.

## 5. Conclusions

The early increase in frequency of PD-1^+^CD8^+^ T cells could be used as an early predictor of favorable prognoses and durable responses in patients with advanced GC receiving cytotoxic chemotherapy.

## Figures and Tables

**Table 1:** Baseline characteristics.

	Increased PD-1^+^CD8^+^ T-Cell Group *(n* = 28)		Decreased PD-1^+^CD8^+^ T-Cell Group *(n* = 40)		
		%		%	*p* Value *
Age (years)					0.234
<65	12	(42.9%)	23	(57.5%)	
≥65	16	(57.1%)	17	(42.5%)	
Sex					0.743
Male	20	(71.4%)	30	(75.0%)	
Female	8	(28.6%)	10	(25.0%)	
ECOG PS					0.298
0–1	25	(89.3%)	39	(97.5%)	
2	3	(10.7%)	1	(2.5%)	
Differentiation					0.074
Well to moderate	12	(42.9%)	9	(22.5%)	
Poor	16	(57.1%)	31	(77.5%)	
HER2					0.999
Positive	2	(7.1%)	4	(10.0%)	
Negative	26	(92.9%)	36	(90.0%)	
Disease status					0.708
Locally advanced	4	(14.3%)	4	(10.0%)	
Metastatic	24	(85.7%)	36	(90.0%)	
Peritoneal seeding					0.684
Yes	14	(50.0%)	22	(55.0%)	
No	14	(50.0%)	18	(45.0%)	
No. of metastatic sites					0.071
≥2	6	(21.4%)	17	(42.5%)	
<2	22	(78.6%)	23	(57.5%)	
CEA (ng/mL)					0.034
>5	14	(50.0%)	10	(25.0%)	
≤5	14	(50.0%)	30	(75.0%)	
CA 19-9 (U/mL)					0.444
>37	8	(28.6%)	15	(37.5%)	
≤37	20	(71.4%)	25	(62.5%)	
Tissue PD-L1					0.182
CPS ≥ 10	11	(39.3%)	19	(47.5%)	
CPS < 10	13	(46.4%)	20	(50.0%)	
Undetermined	4	(14.3%)	1	(2.5%)	

* *p* value from chi-squared tests or Fisher’s exact tests for categorical variables. Data are represented as n (%). ECOG PS, Eastern Cooperative Oncology Group Performance Status; HER2, human epidermal growth factor receptor 2; CEA, carcinoembryonic antigen; CA 19-9, Cancer antigen 19-9; PD-L1, programmed death ligand 1; CPS, combined positive score.

**Table 2:** Univariate and multivariate analysis for survival.

	Univariate			Multivariate		
	HR	(95%CI)	*p* Value	HR	(95%CI)	*p* Value
Overall Survival						
Age (≥65)	1.25	0.73–2.12	0.419			
Sex (male)	1.11	0.60–2.03	0.746			
ECOG PS (2)	1.26	0.75–2.11	0.376			
Differentiation (poorly)	1.23	0.92–1.65	0.157			
HER2 (positive)	1.77	0.69–4.51	0.232			
Disease status (metastatic)	1.37	0.73–2.55	0.328			
No. of metastatic sites (≥2)	3.21	1.83–5.63	<0.001	2.37	1.26–4.44	0.007
CEA (>5 ng/mL)	0.87	0.49–1.53	0.622			
CA 19-9 (>37 U/mL)	1.32	0.76–2.31	0.320			
NLR (≥ median)	2.12	1.23–3.64	0.006	2.37	0.81–2.70	0.206
PLR (≥ median)	1.31	0.77–2.22	0.326			
Increase in PD-1^+^CD8^+^ T-cell frequency	0.43	0.24–0.77	0.004	0.51	0.29–0.92	0.025
Progression-free Survival						
Age (≥65)	1.15	0.70–1.89	0.576			
Sex (male)	1.08	0.61–1.89	0.789			
ECOG PS (2)	1.06	0.64–1.77	0.824			
Differentiation (poorly)	1.13	0.87–1.49	0.359			
HER2 (positive)	1.66	0.71–3.92	0.244			
Disease status (metastatic)	1.53	0.84–2.75	0.161			
No. of metastatic sites (≥2)	3.33	1.89–5.88	<0.001	2.55	1.34–4.86	0.004
CEA (>5 ng/mL)	1.15	0.68–1.94	0.597			
CA 19-9 (>37 U/mL)	1.63	0.97–2.75	0.065	1.16	0.66–2.02	0.609
NLR (≥median)	2.28	1.36–3.84	0.002	1.67	0.93–2.98	0.085
PLR (≥median)	1.3	0.79–2.14	0.305			
Increase in PD-1^+^CD8^+^ T-cell frequency	0.49	0.29–0.83	0.008	0.57	0.33–0.98	0.041

ECOG PS, Eastern Cooperative Oncology Group Performance Status; HER2, human epidermal growth factor receptor 2; CEA, carcinoembryonic antigen, CA 19-9, Cancer antigen 19-9; NLR, neutrophil-to-lymphocyte ratio; PLR, platelet-to-lymphocyte ratio.

## Data Availability

The data presented in this study are available on request from the corresponding author. The data are not publicly available due to institutional restrictions.

## Supplementary Materials

The following supporting information can be downloaded at: https://www.mdpi.com/article/10.3390/cancers15153955/s1, Figure S1: Baseline PD-1^+^CD8^+^ T-cell frequencies among CD8^+^ T cells according to MSI/EBV and HER2 status.; Figure S2: Correlation matrix of laboratory factors; Figure S3: Dynamic change in CD69 and Ki-67 expression of circulatory PD1^+^CD8^+^; Figure S4: Survival difference stratified by the number of metastatic sites (<2 vs. ≥2); Figure S5: Dynamic change in the count and proportion of blood cells from day 0 to day 21; Table S1: The antibodies used in fluorescence-activated cell sorting (FACS); Table S2: HER2, EBV, and MSI status according to increased or decreased frequencies of PD1^+^CD8^+^ T cells; Table S3: Univariate and multivariate analysis for duration of response; Table S4: Baseline characteristics stratified by number of metastatic sites; Table S5: Baseline characteristics according to baseline frequency of PD1^+^CD8^+^ T cells.
