# Supplementary material for: Early Increase in Circulating PD-1+CD8+ T Cells Predicts Favorable Survival in Patients with Advanced Gastric Cancer Receiving Chemotherapy
Source: Cancers (Basel). 2023 Aug 3;15(15):3955. doi: 10.3390/cancers15153955 (PMC10417033; doi:10.3390/cancers15153955)
Supplement: Supplementary file 1 [file cancers-15-03955-s001.zip › Supplemental_Figure_S1.pdf]

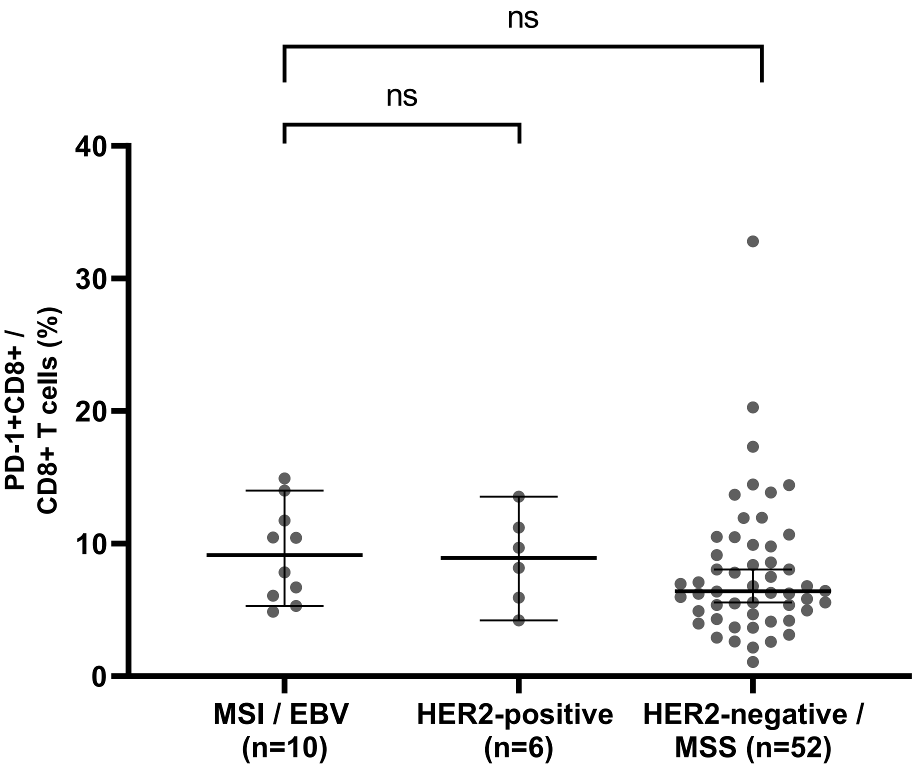

Supplementary Figure S1. Baseline PD-1+CD8+ T-cell frequencies among CD8+ T-cells according to MSI/EBV and HER2 status. No statistical difference was found between the groups. The Mann–Whitney U-test was used for analysis. HER2, human epidermal growth factor receptor 2; EBV, Epstein-Barr virus; MSI, Microsatellite instability; MSS, Microsatellite stable.
