# Supplementary material for: Early Increase in Circulating PD-1+CD8+ T Cells Predicts Favorable Survival in Patients with Advanced Gastric Cancer Receiving Chemotherapy
Source: Cancers (Basel). 2023 Aug 3;15(15):3955. doi: 10.3390/cancers15153955 (PMC10417033; doi:10.3390/cancers15153955)
Supplement: Supplementary file 1 [file cancers-15-03955-s001.zip › Supplemental_Figure_S2.pdf]

a

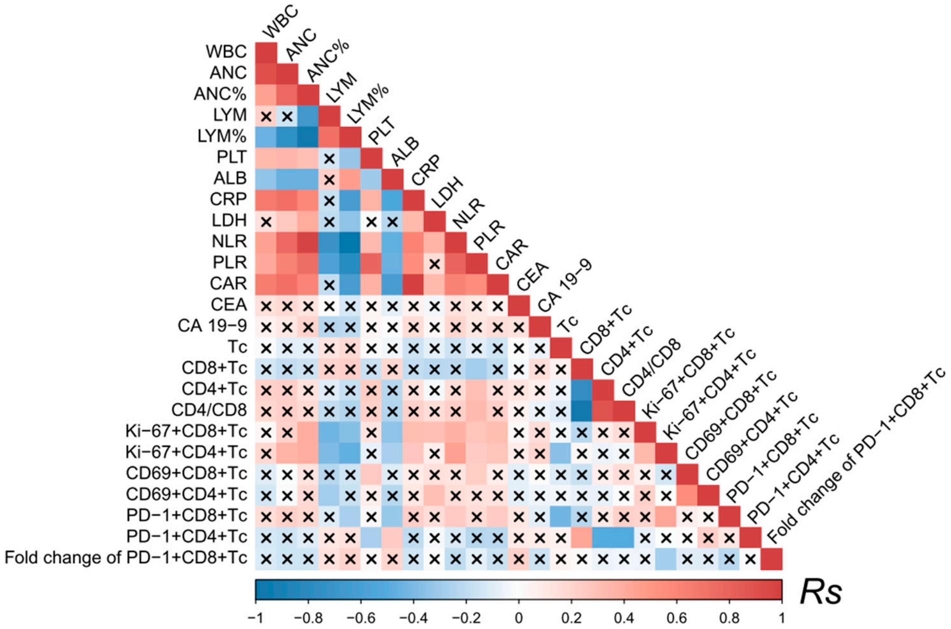

b

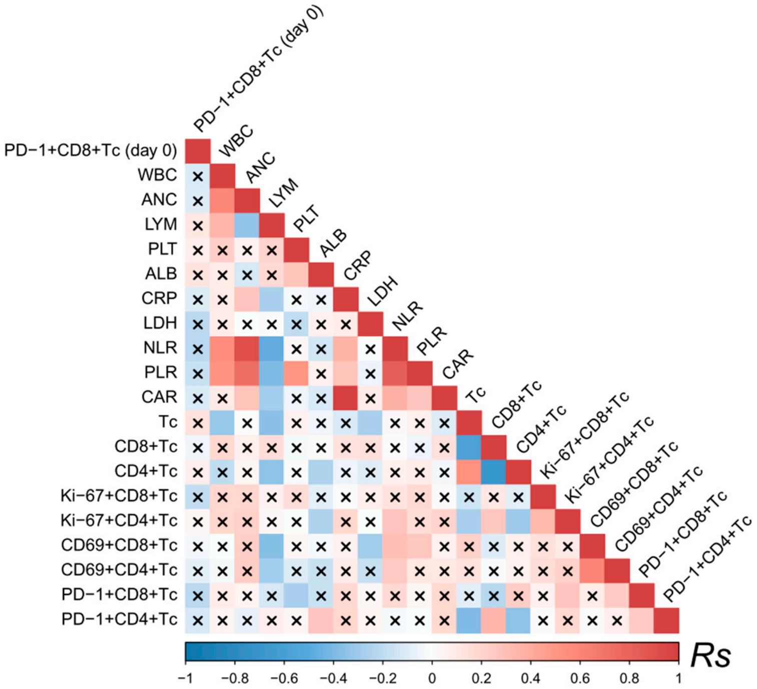

Supplementary Figure S2. Correlation matrix of laboratory factors. (**a**) Correlation matrix between subsets of Tt cells and inflammatory markers from baseline. (**b**) Correlation matrix between the fold changes of subsets of T cells and inflammatory markers from day 0 to day 7. Spearman's correlation analysis was performed. A value of $p < 0.05$ denoted statistical significance. 'x' indicates absence of statistical significance. ***Rs***, Spearman's rank correlation coefficient; WBC, White blood cells; ANC, Absolute neutrophil count; LYM, Lymphocytes; PLT, platelet count; ALB, albumin; CRP, C-reactive protein; NLR, Neutrophil-lymphocyte ratio; PLR, Platelet-lymphocyte ratio; CAR, CRP to albumin ratio; Tc, T cells.
