# Supplementary material for: Early Increase in Circulating PD-1+CD8+ T Cells Predicts Favorable Survival in Patients with Advanced Gastric Cancer Receiving Chemotherapy
Source: Cancers (Basel). 2023 Aug 3;15(15):3955. doi: 10.3390/cancers15153955 (PMC10417033; doi:10.3390/cancers15153955)
Supplement: Supplementary file 1 [file cancers-15-03955-s001.zip › Supplemental_Figure_S3.pdf]

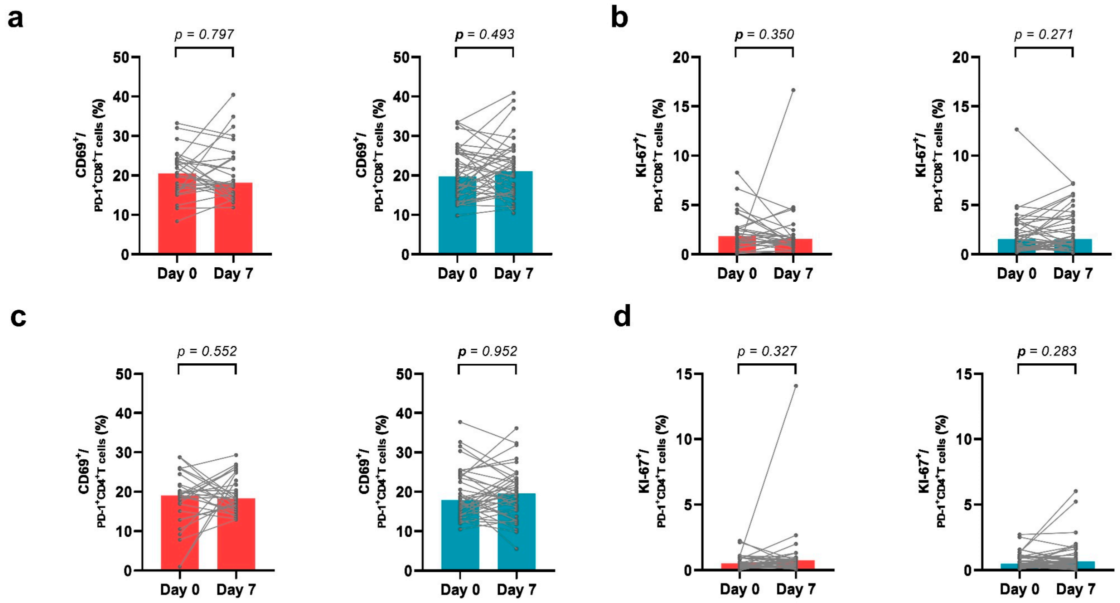

Supplementary Figure S3. Dynamic change of CD69 and Ki-67 expression of circulatory PD1^{+}CD8^{+} (**a**,**b**) and PD1^{+}CD4^{+} (**c**,**d**) T cells from days 0 to 7 in both the increased- and decreased-PD-1^{+}CD8^{+} T-cell groups (red bar and blue bar). A Wilcoxon matched-pairs signed rank test was performed for statistical analysis
