# Supplementary material for: Early Increase in Circulating PD-1+CD8+ T Cells Predicts Favorable Survival in Patients with Advanced Gastric Cancer Receiving Chemotherapy
Source: Cancers (Basel). 2023 Aug 3;15(15):3955. doi: 10.3390/cancers15153955 (PMC10417033; doi:10.3390/cancers15153955)
Supplement: Supplementary file 1 [file cancers-15-03955-s001.zip › Supplemental_Figure_S4.pdf]

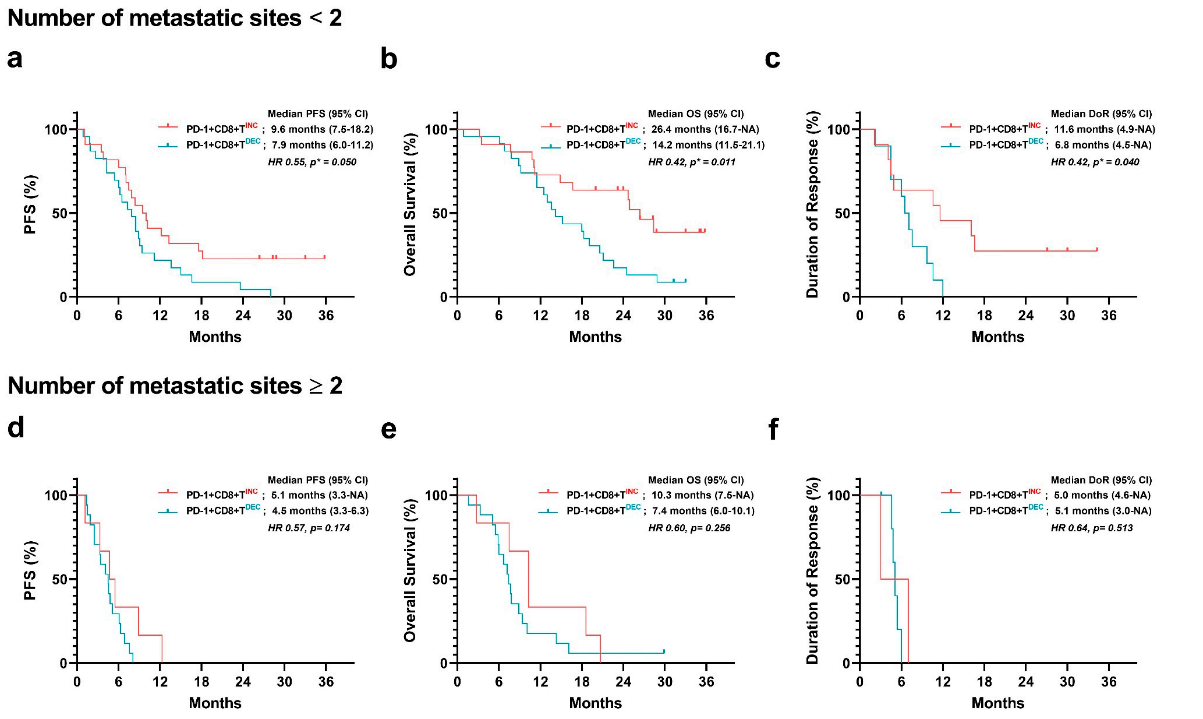

Supplementary Figure S4. Survival difference stratified by the number of metastatic sites (< 2 vs. ≥ 2). (**a–c**) Progression-Free Survival (PFS), Overall Survival (OS) and Duration of Response (DOR) in the increased PD-1$^+$CD8$^+$ T-cell group (n = 22) and the decreased PD-1$^+$CD8$^+$ T-cell group (n = 23) among patients with fewer than two metastatic sites. (**d–f**) PFS, OS and DOR in the increased PD-1+CD8+ T-cell group (n = 6) and the decreased PD-1$^+$CD8$^+$ T-cell group (n = 17) among patients with more than two metastatic sites. The increased PD-1$^+$CD8$^+$ T-cell group showed longer PFS, OS and DOR among patients with fewer than two metastatic sites. (PFS: 9.6 months vs. 7.9 months, p = 0.050; OS: 26.4 months vs. 14.2 months, p = 0.011; DOR: 11.6 vs. 6.8 months, p = 0.040)
