# Supplementary material for: Early Increase in Circulating PD-1+CD8+ T Cells Predicts Favorable Survival in Patients with Advanced Gastric Cancer Receiving Chemotherapy
Source: Cancers (Basel). 2023 Aug 3;15(15):3955. doi: 10.3390/cancers15153955 (PMC10417033; doi:10.3390/cancers15153955)
Supplement: Supplementary file 1 [file cancers-15-03955-s001.zip › Supplemental_Figure_S5.pdf]

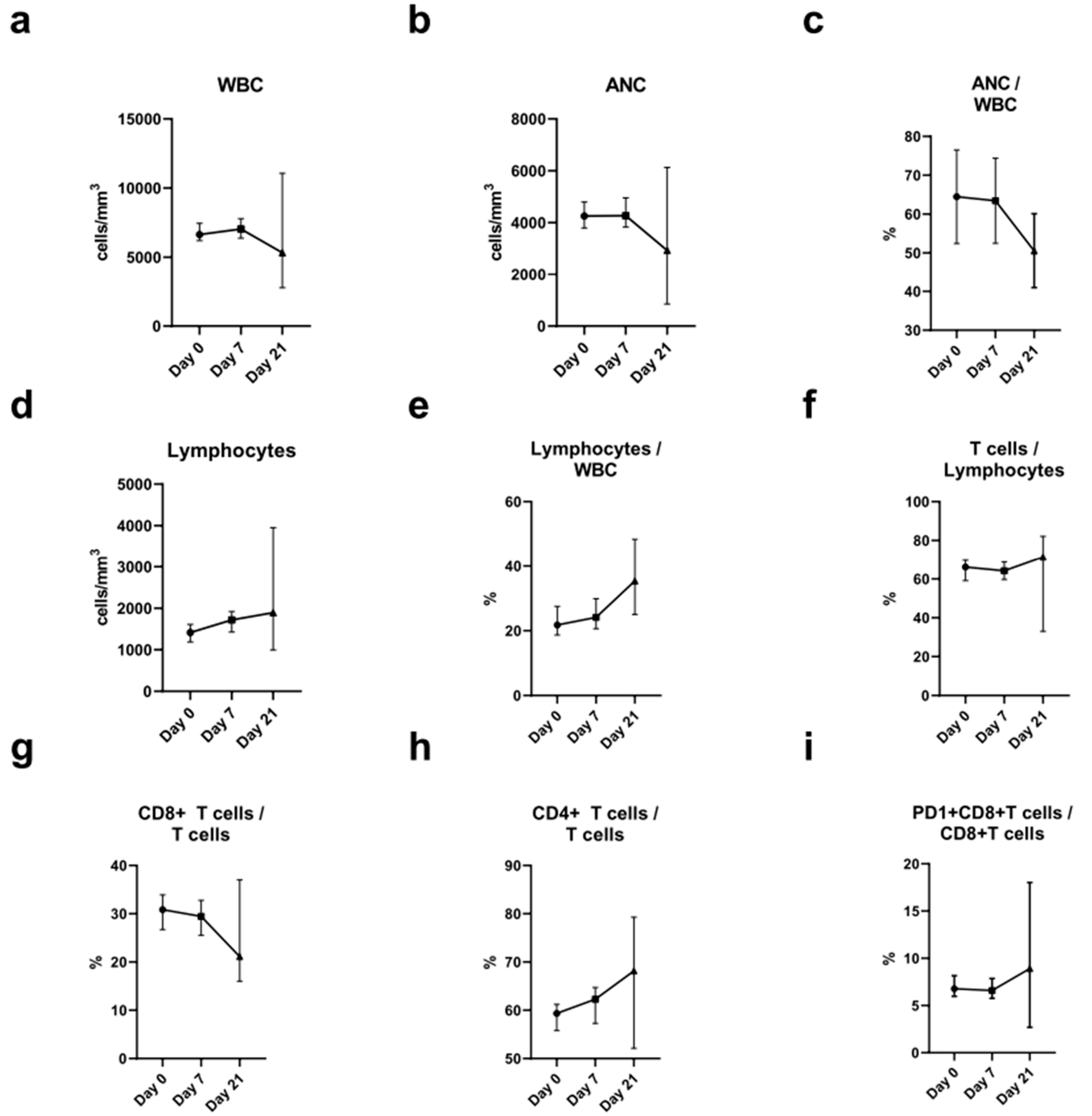

Supplementary Figure S5. Dynamic change in the count and proportion of blood cells from day 0 to day 21. Wilcoxon matched-pairs signed rank test was performed to compare values between day 0 and day 7. The values from Day 21 were not compared with Day 0 and Day 7 due to the low sample size (n = 8). (**a**–**i**) No significant difference was found between day 0 to day 7 in White blood cell (WBC), Absolute neutrophil count (ANC), lymphocytes, the frequencies of T cells among lymphocytes, of CD8+ T cells among T cells, of CD4+ T cells among T cells, and of PD1+CD8+ T cells among CD8+ T cells.
