# Supplementary material for: Early Increase in Circulating PD-1+CD8+ T Cells Predicts Favorable Survival in Patients with Advanced Gastric Cancer Receiving Chemotherapy
Source: Cancers (Basel). 2023 Aug 3;15(15):3955. doi: 10.3390/cancers15153955 (PMC10417033; doi:10.3390/cancers15153955)
Supplement: Supplementary file 1 [file cancers-15-03955-s001.zip › Supplementary Table S1.pdf]

Supplementary Table S1. The antibodies used in fluorescence-activated cell sorting (FACS).

Antibody	Supplier	Cat. No.
**FACS**		
CD3	BioLegend	317314
CD4	BioLegend	357416
CD8	BioLegend	300910
CD45	BioLegend	368510
CD69	BioLegend	310912
Ki-67	BioLegend	151212
PD-1	BioLegend	329908
