# Supplementary material for: Early Increase in Circulating PD-1+CD8+ T Cells Predicts Favorable Survival in Patients with Advanced Gastric Cancer Receiving Chemotherapy
Source: Cancers (Basel). 2023 Aug 3;15(15):3955. doi: 10.3390/cancers15153955 (PMC10417033; doi:10.3390/cancers15153955)
Supplement: Supplementary file 1 [file cancers-15-03955-s001.zip › Supplementary Table S2.pdf]

Supplementary Table S2. HER2, EBV and MSI status according to increased or decreased frequencies of PD1+CD8+ T cells.

	Increased *$PD1^+CD8^+$T-cell group* n=28		*Decreased* *$PD1^+CD8^+$T-cell group* n=40		p value*
HER2					0.999
Positive	2	(7.1%)	4	(10.0%)	
Negative	26	(92.9%)	36	(90.0%)	
EBV					0.999
Positive	2	(7.1%)	4	(10.0%)	
Negative	26	(92.9%)	36	(90.0%)	
MSI					0.638
Instability	1	(3.6%)	3	(7.5%)	
Stability	27	(96.4%)	37	(92.5%)	

* P value from chi-squared tests or Fisher's exact tests for categorical variables. Data are represented as n (%). HER2, human epidermal growth factor receptor 2; EBV, Epstein-Barr virus; MSI, Microsatellite instability.
