# Supplementary material for: Early Increase in Circulating PD-1+CD8+ T Cells Predicts Favorable Survival in Patients with Advanced Gastric Cancer Receiving Chemotherapy
Source: Cancers (Basel). 2023 Aug 3;15(15):3955. doi: 10.3390/cancers15153955 (PMC10417033; doi:10.3390/cancers15153955)
Supplement: Supplementary file 1 [file cancers-15-03955-s001.zip › Supplementary Table S3.pdf]

Supplementary Table S3. Univariate and multivariate analysis for duration of response

	univariate			*multivariate*		
	HR	*(95%CI)*	*p* value	HR	*(95%CI)*	*p* value
**Duration of response (n = 29)**						
Age (≥ 65)	2.03	0.89 - 4.63	0.091	1.07	0.37 - 3.12	0.897
Sex (male)	1.78	0.60 - 5.29	0.297			
ECOG PS (2)	1.26	0.60 - 2.61	0.542			
Differntiation (poorly)	0.76	0.50 - 1.15	0.198			
HER2 (positive)	4.41	1.46 - 13.28	**0.009**	1.57	0.35 - 7.07	0.559
Disease status (metastatic)	0.9	0.31 - 2.62	0.842			
No. of metastatic sites (≥ 2)	3.43	1.28 - 9.18	**0.014**	1.42	0.33 - 6.19	0.639
CEA (>5 ng/mL)	0.79	0.35 - 1.76	0.557			
CA 19-9 (>37 U/mL)	2.05	0.92 - 4.57	0.079	1.59	0.50 - 5.00	0.432
NLR (≥ median)	2.69	1.19 - 6.11	**0.018**	1.95	0.67 - 5.70	0.222
PLR (≥ median)	0.88	0.52 - 2.47	0.754			
Increase in PD1+CD8+T cells	0.38	0.15 - 0.92	**0.033**	0.55	0.18 - 1.63	0.280
