# Supplementary material for: Early Increase in Circulating PD-1+CD8+ T Cells Predicts Favorable Survival in Patients with Advanced Gastric Cancer Receiving Chemotherapy
Source: Cancers (Basel). 2023 Aug 3;15(15):3955. doi: 10.3390/cancers15153955 (PMC10417033; doi:10.3390/cancers15153955)
Supplement: Supplementary file 1 [file cancers-15-03955-s001.zip › Supplementary Table S4.pdf]

Supplementary Table S4. Baseline characteristics stratified by number of metastatic sites.

	No of metastatic sites < 2		*No of metastatic sites ≥ 2*		
	n=45		n=23		p value*
PD-1$^+$CD8$^+$					0.071
Increased	22	(48.9%)	6	(26.1%)	
Decreased	23	(51.1%)	17	(73.9%)	
Age (years)					0.551
< 65	22	(48.9%)	13	(56.5%)	
≥ 65	23	(51.1%)	10	(43.5%)	
Sex					0.596
Male	34	(75.6%)	16	(69.6%)	
Female	11	(24.4%)	7	(30.4%)	
ECOG PS					0.999
0,1	42	(93.3%)	22	(95.7%)	
2	3	(6.7%)	1	(4.3%)	
Differentiation					0.619
Well to moderate	13	(28.9%)	8	(34.8%)	
Poor	32	(71.1%)	15	(65.2%)	
HER2					**0.015**
Positive	1	(2.2%)	5	(21.7%)	
Negative	44	(97.8%)	18	(78.3%)	
Disease status					**0.044**
Locally advanced	8	(17.8%)	0	(0.0%)	
Recurrent or metastatic	37	(82.2%)	23	(100.0%)	
Peritoneal seeding					0.147
Yes	21	(46.7%)	15	(65.2%)	
No	24	(53.3%)	8	(34.8%)	
NLR[†]					**0.021**
≥ 3.2	18	(40.0%)	16	(69.6%)	
< 3.2	27	(60.0%)	7	(30.4%)	
PLR[†]					0.798
≥ 163	22	(48.9%)	12	(52.2%)	
< 163	23	(51.1%)	11	(47.8%)	
CEA (ng/mL)					0.549
> 5	17	(37.8%)	7	(30.4%)	
≤ 5	28	(62.2%)	16	(69.6%)	
CA 19-9 (U/mL)					0.081
> 37	12	(26.7%)	11	(47.8%)	
≤ 37	33	(73.3%)	12	(52.2%)	

Data are n (%). *P value from Chi-square test or Fisher's exact test for categorical variables. HER2=human epidermal growth factor receptor 2. CEA=carcinoembryonic antigen, CA 19-9=Cancer antigen 19-9. PD-L1=programmed death ligand 1. CPS=combined positive score. NLR=Neutrophil to lymphocyte-ratio. PLR=Platelet to lymphocyte-ratio.
