# Supplementary material for: Early Increase in Circulating PD-1+CD8+ T Cells Predicts Favorable Survival in Patients with Advanced Gastric Cancer Receiving Chemotherapy
Source: Cancers (Basel). 2023 Aug 3;15(15):3955. doi: 10.3390/cancers15153955 (PMC10417033; doi:10.3390/cancers15153955)
Supplement: Supplementary file 1 [file cancers-15-03955-s001.zip › Supplementary Table S5.pdf]

Supplementary Table S5. Baseline characteristics according to baseline frequency of PD1+CD8+ T cells

	PD1+CD8+ high (≥ 6.8%) n=34		*PD1+CD8+ low (< 6.8%)* n=34		p value*
**Age (years)**					0.467
< 65	16	(47.1%)	19	(55.9%)	
≥ 65	18	(52.9%)	15	(44.1%)	
**Sex**					**0.012**
Male	30	(88.2%)	20	(58.8%)	
Female	4	(11.8%)	14	(41.2%)	
**ECOG PS**					1.000
0,1	32	(94.1%)	32	(94.1%)	
2	2	(5.9%)	2	(5.9%)	
**Differentiation**					0.793
Well to moderate	10	(29.4%)	11	(32.4%)	
Poor	24	(70.6%)	23	(67.6%)	
**HER2**					0.673
Positive	4	(11.8%)	2	(5.9%)	
Negative	30	(88.2%)	32	(94.1%)	
**Disease status**					0.709
Locally advanced	5	(14.7%)	3	(8.8%)	
Metastatic	29	(85.3%)	31	(91.2%)	
**Peritoneal seeding**					0.145
Yes	15	(44.1%)	21	(61.8%)	
No	19	(55.9%)	13	(38.2%)	
**No. of metastatic sites**					0.442
≥ 2	10	(29.4%)	13	(38.2%)	
< 2	24	(70.6%)	21	(61.8%)	
**CEA (ng/mL)**					1.000
> 5	12	(35.3%)	12	(35.3%)	
≤ 5	22	(64.7%)	22	(64.7%)	
**CA 19-9 (U/mL)**					0.798
> 37	12	(35.3%)	11	(32.4%)	
≤ 37	22	(64.7%)	23	(67.6%)	
**Tissue PD-L1**					0.891
CPS ≥ 10	15	(44.1%)	15	(44.1%)	
CPS < 10	16	(47.1%)	17	(50.0%)	
Undetermined	3	(8.8%)	2	(5.9%)	

* P value from chi-squared tests or Fisher's exact tests for categorical variables. Data are represented as n (%). ECOG PS, Eastern Cooperative Oncology Group Performance Status; HER2, human epidermal growth factor receptor 2; CEA, carcinoembryonic antigen; CA 19-9, Cancer antigen 19-9; PD-L1, programmed death ligand 1; CPS, combined positive score.
